# Metabolic plasticity: an evolutionary perspective on metabolic and circadian dysregulation in bipolar disorder

**DOI:** 10.1038/s41380-025-03123-9

**Published:** 2025-07-19

**Authors:** Iain H. Campbell, Mark A. Frye, Harry Campbell

**Affiliations:** 1 Division of Psychiatry, Centre for Clinical Brain Sciences, University of Edinburgh, Room FU214, Chancellors Building, Royal Infirmary of Edinburgh, Edinburgh, EH16 4SB UK; 2 Professor of Psychiatry, Department of Psychiatry and Psychology, Mayo Clinic, Rochester, MN 55905 USA; 3 Professor of Genetic Epidemiology and Public Health, Centre for Global Health, Usher Institute, University of Edinburgh, Edinburgh, EH16 4UX UK

**Keywords:** Bipolar disorder, Neuroscience

## Abstract

The emerging field of metabolic psychiatry has brought mechanisms of metabolic dysfunction into focus in bipolar disorder research. In this manuscript, we propose that the metabolic features of bipolar disorder provide a new vector from which to understand the role of circadian dysfunction in this condition. A notable feature of bipolar disorder is the photoperiod driven, seasonal occurrence of symptoms and episodes mediated by circadian systems, with mania occurring more frequently in the spring and autumn at times of rapid rate of change in photoperiod, and depression being more prevalent in the winter when photoperiod is attenuated. In this manuscript we note that seasonal adaptations in metabolism are highly conserved evolutionary traits across diverse taxa. Several of the underlying mechanisms mediating seasonal changes in metabolism are conserved in human biology and are implicated in bipolar disorder pathophysiology. Such mechanisms encompass targets of lithium involved in insulin signaling (the phosphatidylinositol cycle, GSK3β and Akt), clock genes (CLOCK and BMAL1), targets of psychiatric and metabolic medications (mTOR and AMPK) and hormonal signaling (melatonin and cortisol). We propose that bipolar disorder may represent a dysregulation of conserved mechanisms of chronometabolic regulation and provide a discussion of the evolutionary context of such mechanisms. Genetic predisposition coupled to novel environmental inputs to human biology including artificial light at night and sustained refined sugar and carbohydrate intake may contribute to states of metabolic and circadian dysregulation in bipolar disorder underlying episodes of mania and depression.

## Introduction: bipolar disorder, metabolism and circadian rhythm

Bipolar disorder was first defined by psychiatrist Emil Kraepelin in his 1921 work “Manic Depressive Insanity and Paranoia”. In this publication Kraepelin noted metabolic and circadian disruption, as well as seasonal variation of symptoms, as particularly notable features of the condition:

*“Repeatedly I saw in these cases moodiness set in in autumn and pass over in spring, when the sap shoots in the trees…”*

*“All these changes indicate that in manic depressive insanity marked disorders of metabolism must take place.”*

*“The attacks of manic-depressive insanity are invariably accompanied by all kinds of bodily changes. By far the most striking are the disorders of sleep and of general nourishment.”* [1]

The past century of research investigating bipolar disorder pathophysiology has brought significant scientific validation to Kraepelin’s early clinical observations of circadian and metabolic dysfunction, establishing these as important features of bipolar disorder.

In this manuscript we highlight the interlinked nature of circadian and metabolic systems, which evolved under selective environmental pressures to mediate seasonal adaptions in metabolic function in response to changes in photo-period and environmental stressors. The highly conserved nature of such mechanisms in human biology and across diverse taxa highlight their essential role in survival. In particular, we discuss the notable overlap of such mechanisms with those of primary interest to bipolar disorder research in the fields of chrono-psychiatry and metabolic psychiatry.

## Seasonal variation of bipolar symptoms

Seasonal variation in bipolar disorder symptoms and episodes is particularly notable among the psychiatric conditions. Systematic review of studies examining seasonality indicate that hospitalisations with episodes of mania peak in the spring and autumn [2] corresponding to times of year with the most rapid change in photo- period around the spring and autumn equinoxes. Conversely, during the winter there is a greater risk of depression in bipolar patients with depressive symptoms reaching a peak during the weeks when photoperiod is at its lowest around the winter solstice [2, 3].

A retrospective population-based study in Taiwan of 15,060 hospital admissions of bipolar disorder patients reported a peak of mania admissions in August and a peak of mixed episodes in March [4]. In a detailed time series analysis of a prospective cohort of 314 people living with bipolar disorder, the most significant peak of manic symptoms occurred around the autumn equinox and the most significant peak of depression around the winter solstice [2]. Hospital admissions for first episode mania in 152 bipolar patients in Korea peaked in the spring (March) and in the autumn (October) [5].

Studies observing a relationship between photo-period and occurrence of mania and depression in bipolar disorder give further scientific grounding to seasonal variation of symptoms. A study examining the relationship of photo-period to hospital admissions with mania in 21,882 patients in New South Wales reported increased admissions in the spring and that rate of change of photo-period was correlated with the number of admissions [6]. And a study of 992 hospital admissions in the United Kingdom reported that hours of daylight and hours of sunshine accounted for 68% of monthly fluctuations in number of admissions for mania [7]. Systematic review and meta-analysis of randomised controlled trials of light therapy indicate some efficacy in bipolar depression which lends further credence to the influence of light exposure on bipolar symptoms [8]. Conversely dark therapy has preliminary indications of effectiveness in bipolar mania [9].

However, seasonal variation is not observed in all studies, and may represent a distinct phenotype in bipolar patients. For example, an analysis of 295 patients in the Finnish Hospital Discharge Register of in-patient admissions, from a northern latitude where variation in solar insolation is more pronounced, found no seasonal variation of mania [10]. A study of 295 patients in India found no seasonal variation of mania, noting the relatively constant climate as a potential factor [11]. And a study of 5317 patients in Canada found no seasonal variation except for mixed state episodes peaking in the summer [12]. There are some indications that a distinct seasonal phenotype may be related to impaired metabolic function. In a study of 1471 outpatients with bipolar disorder, patients with seasonal pattern as compared to those without seasonal pattern, had significantly higher levels of fasting glucose, systolic blood pressure, triglycerides levels, larger abdominal circumference, and a higher body mass index [13].

Overall, systematic review of 51 studies reports evidence for seasonal variation in the majority of studies noting that “Seasonal peaks for different BD mood episodes are observed worldwide and widely replicated”. However, the exceptions noted here indicate that this is not universally observed and may occur in a subset of patients. Taken together, the evidence for increased hospitalisations at times of significant change in photo-period, association between solar insolation and occurrence of episodes, as well as evidence for the effectiveness of light and dark therapy, add scientific plausibility to an influence of seasonal changes in photo- period as a contributing factor in the onset of bipolar symptoms and episodes.

Notably, this is a clear patient priority with several large bipolar support organisations sharing patient experiences of seasonal variation in symptoms and developing and distributing advice on this topic [14–16].

## Seasonal changes in photoperiod and metabolism

Throughout the evolutionary history of biological organisms, seasonal changes in photo-period have driven metabolic adaptations [17, 18]. These adaptations evolved as conserved traits across diverse taxa to optimize energy storage and expenditure in response to selective pressures in the environment. Photoperiod served as a critical environmental cue, enabling organisms to anticipate shifts in temperature, food availability, and reproductive opportunities, and to optimise their conservation and utilisation of energy accordingly.

Shortened photoperiod and reduced light exposure are among the most critical environmental signals triggering the onset of metabolic depression in torpor and hibernation [19, 20]. Reduction in photo-period cues physiological changes orchestrated by the superchiasmatic nucleus (SCN), such as slowed metabolic rate, suppression of circadian rhythms and altered hormonal signalling in preparation for conservation of energy during the winter period. Circadian rhythm and metabolic function are adjusted through changes in clock gene expression and metabolic mechanisms such as insulin signalling which prepare the body for prolonged periods of low metabolic activity.

Seasonal states of hypermetabolism also occur in many species around the time of the rapidly changing photo-period at the spring and autumn equinoxes, associated with survival behaviours such as migration, hunting and reproduction. For example, around the time of the spring and autumn equinoxes, migratory animals living in captivity (and removed from an evolutionary consistent environment), exhibit hyper-arousal, increased metabolic rate, restlessness and insomnia in a phenomenon referred to as “Zugunruhe” [21]. Changes in photoperiod are the most significant environmental cues triggering this phenotype [22]. In controlled environments devoid of natural cues and under conditions of artificial light, the signals triggering the hypermetabolic state can become disrupted leading to less predictable timing of episodes of hyper-arousal.

The central circadian and metabolic mechanisms which mediate seasonal metabolic adaptation are conserved in humans, and remain subject to seasonal variation, albeit attenuated from the more extreme adaptations exhibited in the natural world [23]. In this manuscript we highlight a significant overlap between these mechanisms and those which are of primary interest to bipolar disorder research.

## Conserved mechanisms of seasonal metabolic adaptation and bipolar disorder

Research examining evolutionarily conserved mechanisms from animals to humans has led to several important developments in metabolic research and treatment development. For example, understanding of the role of glucagon-like peptide-1 (GLP1) developed from early observations of the role of exendin-4 in animals leading to the modern class of GLP1RA medications for diabetes and obesity [24]. And the action of insulin was originally studied in animals contributing to the development of modern diabetes treatment [25]. As psychiatry and metabolic science merge in the emerging field of metabolic psychiatry, such research trajectories may prove valuable for identifying and studying metabolic processes in the central nervous system which may be relevant to psychiatric conditions.

For example, changes in clock gene expression such as CLOCK and BMAL1 are a significant focus of chrono-psychiatry research and are also central to research into states of torpor and hibernation [26, 27]. Andrews. et al also highlight adaptations in mitochondrial function, glutamate and GABA, melatonin, and glucose and ketone metabolism in the brain as primary mediators of the torpor phenotype [28]. Giroud et.al highlight mechanisms such as GSK3 phosphorylation, AMPK, adaptations in mitochondrial function, hormonal signaling and inflammation as important mediators of torpor [29].

Here we discuss conserved mechanisms mediating seasonal metabolic adaptation in response to photo-period changes and environmental stressors and their associations with bipolar disorder pathophysiology. The intention of the authors is not to directly extrapolate seasonal metabolic adaptations in evolutionary history to putative roles in the modern diagnosis of bipolar disorder. Rather, we highlight evidence that underlying circadian and metabolic mechanisms have been closely interlinked throughout evolutionary history, and that many of these are conserved in human biology and implicated in bipolar disorder. The connection between these conserved biological mechanisms and those underlying bipolar disorder pathophysiology may represent a useful analogy and evolutionary context for metabolic psychiatry and chrono-psychiatry research as evidenced by the utility of such research trajectories in metabolic science.

## Mechanisms of circadian dysregulation: clock genes, transcriptional translational feedback loops and melatonin

Circadian rhythms are fundamental biological processes which regulate a wide array of physiological functions, including metabolism, sleep-wake cycles and hormone release. Circadian rhythms are governed by an internal clock, primarily located in the suprachiasmatic nucleus (SCN) of the hypothalamus, which orchestrates a range of metabolic and hormonal adaptations in human and animal biology in response to changes in photo-period [30].

Suppression of normal circadian rhythm is a notable feature of both torpor/hibernation states and bipolar disorder, particularly during episodes of depression. In many hibernating species, in response to shortened photo-period in the winter, the robust daily rhythms of activity and rest observed during euthermia are attenuated or suppressed to facilitate extended metabolic depression [26, 31].

During torpor, the SCN adjusts the expression of clock genes involved in transcriptional-translational feedback loops (TTFL) such as CLOCK, BMAL1 and PER2 [26, 32], which are essential for maintaining circadian rhythms. These adaptations facilitate extended reduction of metabolic rate and conservation of energy in response to reduced photo-period and environmental stressors. TTFL mechanisms are widely conserved across species since the earliest forms of life, and represent an important biological mechanism linking photo-period, metabolism and circadian function [33].

Clock genes such as CLOCK and BMAL1 are also a central focus of chrono- psychiatry research in bipolar disorder. Polymorphisms in CLOCK and BMAL1 are associated with bipolar disorder [34, 35] among further metabolic, circadian and genetic factors shared with the torpor/hibernation phenotype summarised in Tables 1 and 2. Abnormalities in melatonin secretion, with delayed or attenuated rhythms, are observed in bipolar disorder patients [36–39] and people with bipolar disorder appear to have hyper-sensitive melatonin suppression in response to light [40]. This is further complicated by the artificial light environment in the modern era which represents a novel input to human biology. A UK Biobank analysis of 87,000 participants reported that increased night-time light exposure (artificially extending photo-period) is associated with increased risk of bipolar disorder [41]. A systematic review analysing data from 1,019,739 individuals from 14 studies reported significantly increased cardiometabolic risk in those who had the greatest night-time exposure to artificial light [42], indicating metabolic consequences of artificial light at night.

**Table 1:** Biological Similarities of Hibernation/Torpor and Bipolar Disorder.

Category	Biological Feature	Role during Torpor/Hibernation	Bipolar Disorder
**Metabolic**	Glucose	Downregulation of glucose metabolism [130]. Downregulation of glycolytic enzymes [131]	Impaired peripheral glucose metabolism [132]. Altered cerebral glucose metabolism [133].
	Metabolic Rate	Reduced, often exceeding 85% suppression [134].	Reduced cerebral metabolic rate in depression. Increased in mania [135]. Significantly reduced physical activity in depression and increased activity in mania [136].
	PI3K/AKT Insulin Signalling	Downregulated PI3K/AKT pathway. Reduced phosphorylation of AKT [78] and GSK3 [137].	Impaired components of insulin signalling. Notably the phosphatidylinositol cycle [70], AKT [93] and GSK3 [138] which are targets of Lithium [70].
	Insulin Resistance	Induced adaptively in peripheral tissues to direct glucose to essential biological functions [130].	More than 50% of bipolar patients have measurable insulin resistance (IR) [102]. IR is associated with rapid cycling and worse clinical outcomes and treatment response [57]. Indications of insulin sensitising medication metformin as therapeutic in bipolar disorder [102].
	Ketone Bodies (e.g., β- hydroxybutyrate)	Increased lipid metabolism in hibernation as adipose tissue is utilised for fuel and glucose metabolism is supressed. Elevated levels of β-hydroxybutyrate [28].	Preliminary indications of a therapeutic effect of ketosis on metabolic and mental health outcomes bipolar disorder [114, 115, 117, 118].
	AMPK	Downregulates metabolic processes in hibernation through interaction with AMPK/PGC-1α/PPAR-α axis [122].	AMPK activators rescue hyperexcitability in neurons from bipolar disorder patients [78].
	Sirtuins	Associated with metabolic and cellular protective mechanisms in torpor [139].	Associated with mood disorders and regulation of circadian rhythm [140]. SIRT1, 2 and 6 mRNA levels reduced in bipolar depression [141].
	mTOR	Supressed mTOR signalling during torpor [91].	mTOR hypoactivity associated with cognitive impairments in bipolar disorder [93].
**Hormonal**	Cortisol	Increased cortisol during hibernation/torpor contributing to reduced metabolic signalling via AMPK [122].	Increased level of cortisol and HPA axis dysfunction [142]. Dysregulated diurnal cortisol pattern [143].
	Melatonin	Supressed plasma melatonin rhythm during hibernation [38, 39].	Supressed melatonin rhythm in bipolar disorder [144].
	Insulin	Increased levels in hyperphagia in preparation for hibernation [145].	>50% of bipolar patients insulin resistant (IR) [102].
	Thyroid Hormones (T3, T4)	Reduced levels to lower metabolic rate [146].	Altered thyroid hormone levels in bipolar disorder [147].
**Immune System and Inflammation**	Immune System	Suppressed immune function, reduction in all circulating leukocytes during hibernation. Reactivation upon arousal [124].	Immune dysfunction. Reduction in circulating leukocytes [148].
	IL-6	Elevated in arousal, reduced in hibernation/torpor [149].	Elevated in mania vs depression [150].
	TNF-α	Elevated in arousal, reduced in hibernation/torpor [151].	Elevated in mania vs depression [152].
**Circadian Rhythm**	Circadian Rhythm	Suppression of circadian systems and extended periods of inactivity during hibernation [153]. Short periods of arousal from torpor later in the day.	Dysfunction of circadian systems and extended periods of inactivity during depression [154]. Eveningness chronotype and delayed sleep phase syndrome are common with waking hours later in the day [154, 155].
	Clock Gene Expression	Altered expression of circadian clock genes to support extended inactivity [26].	Altered expression of clock genes [34, 156].
**Activity**	Psychomotor Activity	Complete or substantial reduction in activity to conserve energy [134].	Reduced activity and psychomotor retardation during depression [157, 158].

**Table 2:** Putative Genetic Factors Common to Torpor and Bipolar Disorder.

Gene	Role in Torpor/Hibernation	Role in Bipolar Disorder
**CLOCK**	Plays a central role in regulation of circadian rhythms in torpor and hibernation [26].	Polymorphisms associated with altered circadian rhythms in bipolar disorder [159] and recurrence of episodes of depression [159].
**BMAL1**	Circadian rhythm regulation during hibernation [26].	Altered expression linked to mood disorders [160].
**NRF2**	Enhanced expression during torpor to regulate antioxidant defence [161].	NRF2 enriched in shared genetic variation between bipolar disorder and chronotype [162].
**FOXO3**	Involved in stress resistance in hibernators [163].	FOXO3a is associated with bipolar disorder [164].
**PPARs**	Increased PPARα mRNA expression during torpor [165].	Some evidence of an association of PPARD with bipolar disorder [166]. Preliminary indications of reduced PPAR-γ and increased prevalence of metabolic syndrome in adolescents with bipolar disorder [167].
**FGF21**	Modulates metabolic processes during fasting in hibernation [168].	FGF21 associated with metabolic effects and treatment response to Valproate in Bipolar Type 2 patients [169].
**HIF1α**	HIF- 1α upregulated during torpor to reduce oxygen dependence and downregulate glucose metabolism [170].	Increased expression of HIF- 1α and HIF- 1β mRNA in bipolar depression [171].
**SIRT1**	SIRT1 downregulated during hibernation regulating gluconeogenesis and thermogenesis [32].	SIRT1, 2 and 6 mRNA levels reduced in bipolar depression [141].

Circadian regulation remains to a degree in daily torpor where states of extended metabolic depression alternate with short periods of hyper-metabolic arousal to allow for necessary survival behaviours [31]. In these states the circadian system remains active but constrains activity to shorter periods to allow for euthermia and foraging to occur [19]. Notably, the metabolic depression of torpor is often induced in the early morning and exited later in the day. The majority of patients with bipolar disorder experience clinically significant sleep disturbance and the tendency is substantially toward eveningness chronotype where patients experience partial and intermittent restoration of energy and function later in the day [43, 44]. For example, this occurs in delayed sleep phase syndrome (DSPS) which is a common form of sleep disruption in bipolar disorder patients [45]. In each case -adaptive in torpor and pathological in bipolar disorder- there is clear decoupling of the synchronization of physiological processes with the external environment.

Torpid animals experience intermittent hyper-metabolic bouts of arousal to allow for foraging and survival behaviours, cycling between metabolic depression and arousal to optimise acquisition of resources and conservation of energy. In patients with bipolar disorder, metabolic dysfunction in the form of insulin resistance and type 2 diabetes is associated with 3-fold risk of rapid-cycling bipolar disorder where sudden shifts between mood states occur in an analogous chrono-metabolic cycle [46].

While bipolar depression and mania are of course distinct phenomena from torpor and arousal states, and do not exhibit all of the features of this phenotype, similar underlying mechanisms mediating metabolic adaptation driven by circadian systems are implicated in both conditions. This may indicate a dysregulation of mechanisms which were once adaptive in the world in which human biology evolved. In the modern era, artificial light, sustained refined carbohydrate/sugar intake and novel environmental stressors provide incongruous inputs to these ancient survival mechanisms which evolved under selective pressure in environments of natural light and relative scarcity.

## Metabolic mechanisms underlying seasonal metabolic adaptation and bipolar disorder

The ability to regulate metabolism in response to signals from the environment has been subject to significant selective pressure, and the mechanisms facilitating metabolic adaptation are therefore among the most highly conserved across time and across diverse taxa. The origins of these mechanisms trace back to some of the earliest forms of life and play a central role in regulating essential physiological processes such as metabolism, growth, reproduction, and aging [47]. For example, insulin signalling pathways are conserved mechanisms from unicellular eukaryotes through to mammals and homo-sapiens. In invertebrates like *Caenorhabditis elegans* and *Drosophila melanogaster*, insulin-like peptides and their receptors have been well characterized, influencing lifespan, development, and stress responses [48–50]. In mammals and homo-sapiens, insulin signalling pathways evolved a higher degree of complexity but their core functionality is remarkably preserved, highlighting important roles in cellular metabolism and energy homeostasis [51]. The conservation of adaptive metabolic mechanisms across diverse taxa and across time emphasize their role in regulating biological processes which are essential to life [52].

Recorded observations of a connection between metabolic dysfunction and bipolar disorder extend back to at least the 18^th^ century and multiple explanations and mechanisms accounting for this connection have been proposed. In 1879 Sir Henry Maudsley noted in “The Pathology of the Mind” that “Diabetes is a disease which often shows itself in families in which insanity prevails” suggesting a mechanism of shared hereditary predisposition [53]. In 1890 George Henry Savage noted in “Insanity and Allied Neurosis” disturbances of glucose metabolism in several of his patients suffering from insanity. He reported these clinical observations in support of Maudsley’s theory, while noting that it was not clear how such metabolic disturbance would directly affect the central nervous system [54]. In 1921 Psychiatrist Ernst Kretschmer proposed an association of “pyknic” body-type characterised by abdominal fat distribution with manic-depression in his work “Physique and Character” [55]. Kretschmer’s later work explored blood chemistry and endocrinology in relation to these constitutional types indicating an interest in underlying biological factors driving this phenotype. Among the first proposals of a direct effect of disrupted glucose metabolism on bipolar disorder were those from C.D. van der Velde and M.W. Gordon in their 1969 manuscript “Manic-depressive illness, diabetes mellitus, and lithium carbonate” published in the Archives of General Psychiatry [56]. The authors reported increased frequency of abnormal glucose tolerance tests in manic patients compared to schizophrenic patients, suggesting impaired glucose metabolism as a possible mechanism linking metabolic function to bipolar disorder.

In recent decades, systematic review and meta-analyses of studies examining prevalence of conditions of metabolic dysfunction in bipolar patients support these early observations, reporting that metabolic dysfunction, in the form of insulin resistance, metabolic syndrome and type 2 diabetes are highly prevalent among people with bipolar disorder [46, 57, 58]. In newly diagnosed patients, rates of metabolic syndrome and insulin resistance are significantly higher than in healthy controls [59]. And metabolic issues persist even when controlling for medication use, indicating an intrinsic role of metabolic dysfunction in bipolar disorder, rather than being simply the result of side effects of medication [59–61]. We note that genetic links between metabolic dysfunction and bipolar disorder are not yet clearly established, for example a recent Mendelian randomization study reported a link between susceptibility to metabolic syndrome and multiple psychiatric conditions, but not bipolar disorder [62]. In contrast, a systematic review of genome wide and candidate gene studies identified 24 Cardiometabolic Mood Disorders hub (CMMDh) genes shared between mood disorders (including bipolar disorder) and cardiometabolic diseases [63]. Significant relationship between BMI and genetic risk factors for major depressive disorder were recently reported in a large genome-wide association study and further research of this kind is needed to better understand genetic risk factors for bipolar disorder and their relationship to metabolic parameters [64].

Here we discuss specific metabolic mechanisms which represent significant research loci in bipolar disorder and are implicated in conserved mechanisms of seasonal metabolic adaptation.

## Role of the phosphatidylinositol cycle in the phosphatidylinositol 3 kinase/protein kinase B (PI3K/Akt) insulin signalling pathway

The most notable metabolic adaptation in states of hibernation and torpor is the transition to energy-conserving states of reduced basal metabolic rate and a switch from reliance on glucose to utilisation of adipose tissue and fatty acid metabolism [65–67] facilitated by insulin signalling mechanisms. Insulin signalling pathway activation has been observed to reduce fivefold in skeletal muscle during torpor in a non-human primate compared with aroused state [65] and forty-nine fold in avian species during torpor [68]. When the PI3K/Akt insulin signalling pathway is downregulated, energy is conserved by reduced glucose transport and metabolism. Pyruvate dehydrogenase (PDH), an enzyme which converts glucose derivative pyruvate into TCA cycle intermediate acetyl-CoA, is tightly regulated by kinases (PDK), which phosphorylate and inactivate PDH, thereby inhibiting glucose metabolism. This metabolic shift favours the utilization of fatty acids over glucose oxidation. The PI3K/Akt pathway is preserved in humans and acts via regulation of pyruvate dehydrogenase (PDH) and it’s kinases (PDKs), playing a central role in metabolic adaptation to environmental cues [69].

The PI3K/Akt pathway and downstream targets encompass several mechanisms of central interest to bipolar disorder research including the primary targets of Lithium: the phosphatidylinositol cycle and GSK3β, as well as closely interlinked metabolic mechanisms Akt, mTOR and AMPK [70, 71]. The phosphatidylinositol cycle is a focus of significant research into the mechanism of action of Lithium and generates second messengers such as diacylglycerol (DAG) and phosphatidylinositol 3,4,5 triphosphate (PIP3) which are important mediators of insulin signaling to diverse metabolic systems. In response to insulin stimulation, PI3K phosphorylates phosphatidylinositol 4,5 bisphosphonate (PIP2) to generate phosphatidylinositol 3,4,5 triphosphate (PIP3) which activates Akt. In turn Akt phosphorylates Ser21 of GSK3α and Ser9 of GSK3β resulting in inhibition of GSK3β [70, 72]. This pathway connects metabolic status to environmental and nutritional signals mediated by insulin. Lithium also leads to inhibition of GSK3β through competitive inhibition at the binding site of the GSK3 cofactor magnesium [73, 74]. We have proposed that the effects of lithium may therefore be partly explained through its effects on insulin signalling pathways and have noted that lithium has demonstrated effects on glucose metabolism and insulin resistance [70, 75–77]. A recent study reported findings supporting this perspective in neurons derived from bipolar patients [78]. The study reported that lithium upregulated the insulin signalling Akt pathway in neurons from lithium responsive bipolar patients and that an activator of Akt reproduced similar effects to Lithium in reducing hyperexcitability in BD neurons.

By modulating insulin signaling pathways in varying degrees, humans and torpid animals may optimise the use of glucose towards essential survival functions, maintain metabolic stability, preferentially utilise adipose tissue as an energy reserve and ensure survival during prolonged periods of fasting. The average human body can store around 2400 calories derived from glucose in glycogen stores and well in excess of 100,000 calories in adipose tissue [79, 80]. Therefore, under conditions of scarcity, mechanisms which optimise the utilisation of available glucose toward essential survival functions in the CNS and upregulate fatty-acid metabolism are important for survival. The evolutionary function of insulin resistance was therefore adaptive in nature. During periods of fasting, insulin signalling pathways are modulated, causing peripheral muscle tissue to become insulin resistant and reduce glucose uptake in order to channel available glucose to more essential functions of the central nervous system (CNS) [81]. In a pre-agricultural and evolutionarily consistent environment, with periods of scarcity, this state served to preferentially direct glucose away from muscle tissue and towards more essential survival functions in the CNS. In the modern environment of artificial light and year-round refined carbohydrate intake, dysregulation of this adaptive mechanism may create a state of metabolic dysfunction in both peripheral tissues and the CNS.

The switch between glycolytic metabolism and fatty-acid metabolism facilitated by insulin signalling mechanisms is among the most significant seasonal metabolic adaptations. This is most explicitly exemplified by the visible storage of adipose tissue in the pre-winter period and utilisation of adipose tissue for metabolic fuel during winter hibernation and torpor. However, this adaptation also occurs throughout the year in response to stress, and threats in the environment such as illness, sepsis or starvation. During such times adipose tissue served as a reliable metabolic fuel to carry life through times of scarcity or stress when carbohydrate intake was not guaranteed. This is demonstrated for example by the increased expression of hormone GDF15 which conveys somatic distress in response to sepsis, starvation and other stressors, and subsequent GDF15 mediated induction of lipid metabolism and ketogenesis [82, 83]. In other words, diverse forms of life have evolved a survival mechanism which transitioned the body to a state of metabolic depression, energy conservation and fatty-acid metabolism to increase the chances of survival in reaction to environmental stressors and seasonal scarcity. It is possible that such adaptive mechanisms, many of the components of which are subject to modification by lithium, may become dysregulated in a modern environment and contribute to an analogous state of metabolic depression in bipolar patients.

## GSK3

Glycogen synthase kinase 3 (GSK3) plays a significant downstream role in PI3K/Akt insulin signalling through phosphorylation via Akt. GSK3 is a serine/threonine kinase with two isoforms, GSK3α and GSK3β, both highly expressed in the brain.

GSK3 regulates glycogen synthesis, circadian rhythms, neuroplasticity, and inflammation and its activity is influenced by light-mediated signals via the SCN [84]. Research indicates that GSK3β phosphorylation oscillates with the circadian cycle, affecting clock proteins like PER2 and CRY2, which are mediators of hibernation and torpor [85].

GSK3β activity is subject to significant modification during states of metabolic depression in torpor [86]. The phosphoprotein abundance of GSK3β increases around 5-fold in the brain in deep torpor compared to normal levels observed in the summer. This adaptation occurs both seasonally and in response to torpor and arousal bouts throughout the year [87]. Increases in GSK3β in torpor occur alongside increased phosphoprotein abundance of pyruvate dehydrogenase indicating adaptations in GSK3 β mediated insulin signaling [86].

In peripheral blood mono-nuclear cells (PBMCs) of bipolar disorder patients, GSK3 activity is dysregulated [88] and insulin stimulation causes robust increase in phosphorylated GSK3β in lithium responsive patients in contrast to decreases in lithium non-responsive patients [89]. GSK3 is elevated during mania and this is mitigated by increased serine phosphorylation by lithium [88]. Conversely, reduced total GSK3β distinguishes patients with bipolar depression from those with unipolar depression [90]. The dynamic dysregulation of GSK3 differentiating lithium responsive and non-responsive patients in response to insulin stimulation may indicate a role of GSK3 in insulin signalling in bipolar disorder, paralleling this function in the torpor phenotype.

GSK3 mediates adaptations in circadian and metabolic processes in response to photo-period and is one of the most studied mechanisms in bipolar disorder research due to its implication in the mechanism of action of lithium. The role of GSK3 in states of hibernation and torpor highlights its evolutionary preservation and function at the intersection of metabolic and circadian processes.

## mTOR

A further mechanism intricately interlinked with insulin signalling and GSK3 activity is the mechanistic target of rapamycin (mTOR) pathway which integrates nutrient and energy signals to regulate cell growth, synaptic plasticity, and circadian rhythms. mTOR activity fluctuates with nutrient availability and environmental cues and is heavily suppressed during metabolic depression in torpor and increased during arousal from torpor [91]. In bipolar depression reduced mRNA expression in the AKT1/mTOR pathway has been observed [92] and mTOR hypoactivity contributes to altered synaptic plasticity, mood instability and cognitive impairment [93]. In peripheral immune cells from bipolar patients, dynamic increases in phosphorylated mTOR in response to insulin stimulation indicate a form of metabolic dysregulation which may be associated with response to lithium [89].

Circadian clocks regulate mTOR signalling during calorie restriction (e.g., during winter hibernation/torpor), where mTOR signalling is suppressed, promoting autophagy and energy conservation. Conversely, in times of abundance (e.g., spring/summer), mTOR activation supports anabolic processes like protein synthesis and neurogenesis [94]. In rats increased mTOR and AKT1 activity has been observed in the brain during mania-like behaviour [95]. And rapamycin, an mTOR inhibitor reduces mania-like behaviour in animals [96]. The PI3K/Akt/mTOR pathway is also an important target associated with the effects of multiple pharmacological agents utilised in psychiatry including olanzepine, ketamine and lithium [89, 97, 98].

mTOR is a metabolic target of increasing interest in psychiatric research due to its implication in multiple psychiatric conditions and the action of pharmacological agents. Here we note its role in both seasonal metabolic adaptation and bipolar disorder, in the context of its function at the intersection of metabolic and circadian systems.

## AMP-activated protein kinase (AMPK)

AMPK is a regulator of cellular energy homeostasis, activated under conditions of low energy (high AMP/ATP ratio) and plays a significant role in the metabolic adaptation to states of metabolic depression. AMPK promotes energy conservation by inhibiting anabolic processes (e.g., via mTOR suppression) and enhancing catabolic pathways such as fatty acid oxidation. During hibernation phosphorylation of AMPK is enhanced throughout the brain indicating a role of AMPK mediated metabolic adaptation [99, 100]. AMPK is also intricately interlinked with circadian mechanisms facilitating seasonal adaptation, modulating clock component cryptochrome 1 (CRY1) [101]. In bipolar disorder, cerebral AMPK activity is implicated in neuronal excitability. In a recent study an AMPK activator reduced hyperexcitability in lithium responsive neurons [78] and this effect was mediated by Akt signalling, implicating the insulin signalling pathway. There are some early indications that modulating AMPK may be beneficial in psychiatric conditions. For example, an RCT of metformin in bipolar patients - an activator of AMPK - reported improvements in metabolic and psychiatric symptoms alongside reversal of insulin resistance [102].

AMPK is a critical regulator of metabolic adaptation in torpor and hibernation and emerging evidence indicates a role in neuronal excitability and mood regulation in bipolar disorder. During episodes of depression, which often peak in winter, AMPK overactivation may mimic energy-conserving states, leading to reduced neuronal activity and anhedonia.

## Ketosis

In response to the described circadian and metabolic adaptations during metabolic depression, the body and brain transition from reliance on glycolytic metabolism to fatty acid metabolism facilitated by ketosis. Ketosis is an important metabolic adaptation for many species that undergo hibernation and torpor [103]. During periods of reduced metabolic activity, species rely on their fat reserves for energy [28]. The liver converts these fat stores into ketone bodies, such as β- hydroxybutyrate and acetoacetate, which can be used as an efficient energy source by tissues including the brain and muscle. In many hibernating animals, the shift from glucose to ketone metabolism is a fundamental component of their physiological adaptations [104, 105]. Throughout the hibernation period of metabolic depression, which can last several months, ketosis ensures a steady alternative supply of energy from adipose tissue, supporting vital bodily functions, while minimizing breakdown of skeletal muscle.

Torpor, the state of short-term metabolic depression observed in mammals, also leverages ketosis for energy management but can occur throughout the year in response to environmental stressors [106]. During torpor, an animal’s body temperature and metabolic rate drop significantly, reducing energy expenditure. However, a basal level of energy to maintain essential physiological processes is required and ketosis provides this energy efficiently, producing 31% more ATP per molecule of oxygen than pyruvate, the end product of glycolysis [107].

In an evolutionarily consistent environment, adaptations in insulin signalling induced adaptative insulin resistance and downregulation of glucose metabolism to conserve available glucose for the central nervous system and to utilise ketone bodies as the primary energy substrate. By shifting the body’s primary energy source from glucose to ketone bodies, ketones can bypass insulin-resistant pathways and sustain metabolic function even during states of profound metabolic depression.

Perhaps the most significant adaptation in states of metabolic depression in animals is the substantial inhibition of the pyruvate dehydrogenase complex (PDC), the primary rate-limiting step in glucose oxidation. In humans, in conditions where pathological inhibition of PDC occurs such as in Pyruvate Dehydrogenase Deficiency and Leigh Syndrome, the primary expression of pathology is neurological (seizure) and induction of ketosis through a ketogenic diet is the standard of care [108].

**Fig. 1:**
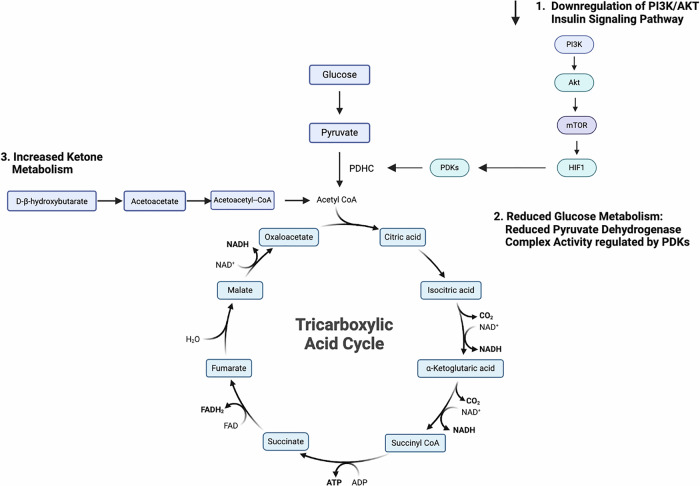
Ketone Bodies Provide an Alternative Metabolic Substrate in Metabolic Depression During Hibernation/Torpor and States of Insulin Resistance. GSK3 - Glycogen Synthase Kinase 3, mTOR - Mechanistic Target of Rapamycin, AMPK - AMP-activated Protein Kinase, HIF-1α - Hypoxia-Inducible Factor 1-alpha, IL-6 - Interleukin 6, TNF-α - Tumor Necrosis Factor-alpha, CLOCK - Circadian Locomotor Output Cycles Kaput, BMAL1 basic helix-loop-helix ARNT like 1, HIF1A- Hypoxia-Inducible Factor 1-alpha.

Ketone bodies such as beta-hydroxybutyrate do not rely on flux through the PI3K/AKT regulated pyruvate dehydrogenase complex (PDC) to generate energy in the citric acid cycle and enter through an alternative pathway via conversion to Acetyl-CoA bypassing the PDC [109] as illustrated in Fig. 1.

The neurological effects of ketosis were first observed by Russell Wilder and Mynie Peterman at the Mayo Clinic in fasted epilepsy patients [110]. However, it was ascertained that ketosis and seizure reduction could also be achieved through increasing fatty-acid metabolism through a ketogenic diet leading to 13 RCTs and over 100 years of clinical application in epilepsy [111]. In contrast to fasting, a ketogenic diet provides ketone bodies while also signalling an abundance of calories, allowing normal metabolic rate to be sustained even under conditions of impaired insulin signalling and metabolic depression.

It is likely that early humans entered periods of ketosis and fatty acid-based metabolism several times a year in response to selective pressures in the environment and changes in food availability. In the post-agricultural era, where access to high carbohydrate foods has become increasingly available, states of established ketosis have become infrequent. Metabolic mechanisms, exposed on a continual basis to hyper-stimuli, begin to break down and disrupt metabolic homeostasis. For example, phosphorylation of components of the insulin signalling pathway such as Akt and GSK3 becomes blunted under such conditions [112]. The preliminary observations of beneficial effects of ketosis in psychiatric conditions from case series, pilot studies, and observational studies may be partially explained by the implication of these mechanisms of disrupted metabolic adaptation in bipolar disorder [113–120]. The findings in this area are preliminary, and over 20 clinical trials are now in progress to further investigate the effects of induction of ketosis in neuropsychiatric conditions beyond epilepsy. Several useful treatments for bipolar disorder including lamotrigine, valproate and carbamazepine have come from epilepsy research. And recent observations of effects of ketosis on brain glutamate metabolism in bipolar patients [113] -a putative mechanism of action of epilepsy medications such as lamotrigine – make this an interesting research trajectory for further investigation.

## Hormonal and immune mechanisms and biomarkers

Adaptations in hormonal signaling, orchestrated by the SCN play a significant role in coordinating seasonal metabolic adaptation. Melatonin is regulated by light-dark cycles, with secretion being decreased during longer daylight periods (e.g., spring/summer), promoting activity and energy expenditure, and increased in winter to support energy conservation by reducing metabolic rate, as observed in hibernating animals [121]. Melatonin is also a hormone of central interest to chrono-psychiatry research. In BD, melatonin dysregulation disrupts circadian rhythms, contributing to sleep disturbances and mood episodes [37]. Cortisol, a stress hormone, also fluctuates seasonally, rising in winter to mobilize energy stores under scarcity, modulated by the hypothalamic-pituitary-adrenal (HPA) axis [122]. Cortisol dysregulation is commonly observed in bipolar patients, and elevated cortisol has been observed to correlate with symptoms of depression [123].

In hibernation the immune system is suppressed with significant reduction of circulating leukocytes [124]. During arousal, monocytes and neutrophils increase rapidly [124]. In BD, immune dysregulation is well established, with elevated IL-6, TNF-α, and C-reactive protein (CRP) levels during mood episodes [125]. Seasonal variation of systematic inflammation appears to be greater in bipolar patients [126] and mania is associated with an acute inflammatory state [127].

Bipolar depression may represent a state of dysregulated inflammation coupled with functional immune impairments, with indications of both increased inflammatory markers such as IL-6 and IL-8 [128] and also elements of immune suppression indicated by lower levels of IL-4 [129].

**Fig. 2:**
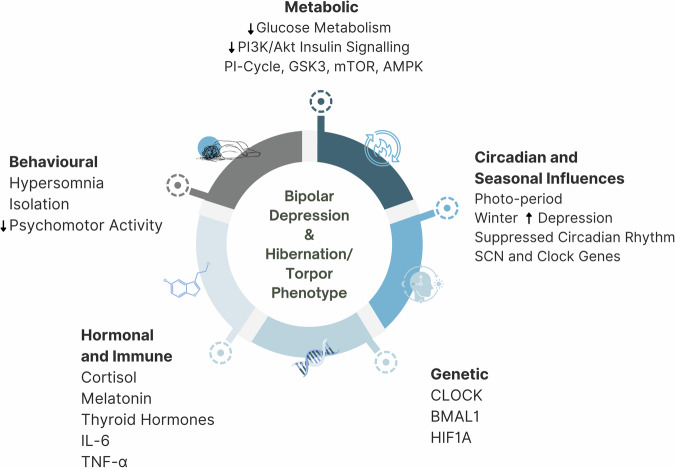
Mechanisms and Biomarkers Shared between the Torpor/Hibernation Phenotype and Bipolar Depression. PI3K - Phosphoinositide 3-Kinase, AKT - Protein Kinase B, mTOR - Mechanistic Target of Rapamycin, HIF1 - Hypoxia-Inducible Factor 1, PDHC - Pyruvate Dehydrogenase Complex, PDKs - Pyruvate Dehydrogenase Kinases, D-β-Hydroxybutyrate - D-Beta-Hydroxybutyrate, Acetoacetate - Acetoacetate, Acetoacetyl-CoA - Acetoacetyl Coenzyme A, NAD + - Nicotinamide Adenine Dinucleotide (oxidized form), NADH – Nicotinamide Adenine Dinucleotide (reduced form), FAD - Flavin Adenine Dinucleotide, FADH2 - Reduced Flavin Adenine Dinucleotide, ATP - Adenosine Triphosphate, ADP - Adenosine Diphosphate.

A summary of the metabolic mechanisms shared between seasonal metabolic adaptation and bipolar disorder are detailed in Fig. 2.

## Metabolic plasticity

In addition to the observation of metabolic, circadian and seasonal aspects of bipolar disorder, Emil Kraepelin highlighted in *Manic Depressive Insanity and Paranoia* a proposal from a colleague that the metabolic features and periodic occurrence of bipolar disorder may be explained by an evolutionary perspective:

*“Stransky also searches for an explanation of manic-depressive insanity from the point- of view of metabolic disorders…[he] emphasizes the ancestral relations between emotional life and periodicity.”* [1]

The clinical observations of Kraepelin of circadian and metabolic dysfunction in bipolar disorder have been well substantiated over the past century of chronopsychiatry, multiomics, brain imaging and metabolic research elucidating mechanisms of disrupted sleep architecture, seasonality of symptoms, and metabolic and circadian dysregulation. At the mechanistic level this is expressed as dysregulation of circadian and metabolic signalling networks implicating several targets of psychiatric medications such as GSK3β, the phosphatidylinositol cycle, Akt, and mTOR, clock genes active in the SCN such as CLOCK and BMAL1 and dysregulation of hormones such as melatonin and cortisol.

Long preceding the modern diagnosis of bipolar disorder, adaptive chronometabolic mechanisms evolved under selective pressures throughout the evolutionary history of mammalian life where interconnection between circadian regulation and metabolic function were essential for survival. An important aspect of the function of conserved chronometabolic mechanisms was to adapt conservation and utilisation of energy in response to environmental signals. In particular, such mechanisms facilitated seasonal metabolic adaptations between glycolytic and fatty-acid metabolism which allowed life to survive on energy stored as adipose tissue when resources were scarce in the winter and when stressors such as illness or sepsis prevented resource acquisition.

In the modern era, artificial light conditions and continuous year-round refined carbohydrate and sugar consumption provide incongruous inputs to these ancient, conserved systems of circadian and metabolic regulation, contributing to the high prevalence of circadian and metabolic dysfunction in bipolar patients. The breakdown of these adaptive chronometabolic mechanisms may result in a hypometabolic state *analogous* to the torpor phenotype. The torpor-like state in bipolar depression is characterised by impaired insulin signalling (implicating PI- Cycle, GSK3, mTOR and AMPK), impaired cerebral glucose metabolism, suppression of clock genes (CLOCK and BMAL1) and circadian rhythm, dysregulation of the immune system (IL-6, IL-4, CRP) and altered hormonal signalling (dysregulated melatonin and cortisol secretion). There are also *analogous* features shared between seasonal hyper-metabolic states, arousal states, and bipolar mania where circadian and metabolic mediators such as CLOCK and mTOR play a key role. We have noted that mania peaks around the spring and autumn equinoxes when photo-period changes most rapidly and during this time many species experience hypermetabolic states driven by chronometabolic systems. At these times of year manic patients exhibit degrees of insomnia, hyperactivity, increased energy, disrupted circadian rhythm and hypersexuality, in a pathological state analogous to similar behavioural traits occurring across many species at these times of year.

It is important to note that despite the similarities noted here, seasonal metabolic adaptations in animals and bipolar depression/mania are distinct phenomena. For example, the significant reduction of body temperature in torpid animals is not necessarily present in bipolar depression, and bipolar mood states, while influenced by environmental factors such as photoperiod are subject to wider influences and occur with less predictable triggers. The explanatory scope of seasonal metabolic adaptation is therefore does not encompass the totality of bipolar clinical presentation and pathophysiology. With this being clearly stated, we note that seasonal variation of bipolar symptoms requires a biological explanation and metabolic adaptations are among the most established seasonal variations across diverse forms of life. Several of the underlying circadian and metabolic mechanisms mediating metabolic adaptation to environmental cues are preserved in humans and are implicated in bipolar disorder. We propose, therefore, that bipolar disorder may represent a condition of dysregulation of evolutionarily conserved mechanisms of chronometabolic regulation. This may occur at the intersection of genetic predisposition and interaction with hyper-stimuli from an artificial light and food environment damaging chronometabolic mechanisms. For example, sustained refined sugar and carbohydrate intake impairs phosphorylation of key component of insulin signaling networks such as GSK3 and Akt [112]. In this manuscript we have described several of the core mechanisms and their links to existing treatment modalities such as lithium, olanzepine, light and dark therapy and circadian interventions.

The implication of these chronometabolic mechanisms in bipolar disorder may also provide context for the preliminary indications of effects of metabolic treatment strategies which are the focus of the emerging field of metabolic psychiatry [102, 113–115] and circadian treatment modalities which are already utilised in clinical care. Given this context, research in metabolic psychiatry may consider advancing research trajectories at the intersection of conserved circadian and metabolic mechanisms, with a view to development of new chronometabolic treatment modalities addressing psychiatric symptoms and the considerable metabolic and circadian co-morbidities present in bipolar disorder. We propose than insulin signaling mechanisms represent a promising frontier for research given their connection to seasonal metabolic adaptation, the primary targets of lithium, and demonstrated role in states of metabolic dysfunction in multiple-organ systems including the brain and CNS. Investigation of insulin sensitising medications, incretin-based treatments, modulation of clock gene function, specific targeted phosphorylation of components of PI3K/Akt insulin signalling and translation of neurometabolic modalities such as ketosis are several avenues available for investigation in metabolic psychiatry research.

## References

[1] Lord JR. Manic-depressive Insanity and Paranoia. By Prof. Emil Kraepelin; translated by R. Mary Barclay, M.A., M.B.; edited by George M. Robertson, M.D., F.R.C.P.Edin. Edinburgh: E. & S. Livingstone, 1921. Demy 8vo. Pp. 280. Forty- nine illustrations, eighteen in colour. Price 12s. 6d. J Ment Sci. 1921;67:342–6.

[2] Akhter A, Fiedorowicz JG, Zhang T, Potash JB, Cavanaugh J, Solomon DA, et al. Seasonal variation of manic and depressive symptoms in bipolar disorder. Bipolar Disord. 2013;15:377–84. 23621686 10.1111/bdi.12072 PMC3731411

[3] Della DF, Allison S, Bidargaddi N, Wa SK, Bastiampillai T. An umbrella systematic review of seasonality in mood disorders and suicide risk: the impact on demand for primary behavioral health care and acute psychiatric services. Prim Care Companion CNS Disord. 2023;25:22r03395. 37230063 10.4088/PCC.22r03395

[4] Lee HC, Tsai SY, Lin HC. Seasonal variations in bipolar disorder admissions and the association with climate: A population-based study. J Affect Disord. 2007;97:61–9. 16890994 10.1016/j.jad.2006.06.026

[5] Lee HJ, Kim L, Joe SH, Suh KY. Effects of season and climate on the first manic episode of bipolar affective disorder in Korea. Psychiatry Res. 2002;113:151–9. 12467954 10.1016/s0165-1781(02)00237-8

[6] Parker G, Hadzi-Pavlovic D, Bayes A, Graham R. Relationship between photoperiod and hospital admissions for mania in New South Wales, Australia. J Affect Disord. 2018;226:72–6. 28964995 10.1016/j.jad.2017.09.014

[7] Suhail K, Cochrane R. Seasonal variations in hospital admissions for affective disorders by gender and ethnicity. Soc Psychiatry Psychiatr Epidemiol. 1998;33:211–7. 9604670 10.1007/s001270050045

[8] Lam RW, Teng MY, Jung YE, Evans VC, GoZlieb JF, Chakrabarty T, et al. Light therapy for patients with bipolar depression: systematic review and meta- analysis of randomized controlled trials. Can J Psychiatry Rev Can Psychiatr. 2020;65:290–300. 10.1177/0706743719892471 PMC7265610 31826657

[9] Barbini B, BenedeZi F, Colombo C, Dotoli D, Bernasconi A, Cigala-Fulgosi M, et al. Dark therapy for mania: a pilot study. Bipolar Disord. 2005;7:98–101. 15654938 10.1111/j.1399-5618.2004.00166.x

[10] Partonen T, Lönnqvist J. Seasonal variation in bipolar disorder. Br J Psychiatry J Ment Sci. 1996;169:641–6. 10.1192/bjp.169.5.641 8932896

[11] Jain S, Kaliaperumal VG, ChaZerji S, Rao S, Murthy RS. Climate and admissions for mania in the tropics. J Affect Disord. 1992;26:247–50. 1479137 10.1016/0165-0327(92)90102-c

[12] Whitney DK, Sharma V, Kueneman K. Seasonality of manic depressive illness in Canada. J Affect Disord. 1999;55:99–105. 10628878 10.1016/s0165-0327(98)00197-9

[13] Geoffroy PA, Godin O, Mahee D, Henry C, Aubin V, Azorin JM, et al. Seasonal paZern in bipolar disorders and cardio-vascular risk factors: a study from the FACE-BD cohort. Chronobiol Int. 2017;34:845–54. 28537802 10.1080/07420528.2017.1324472

[14] Seasons and Cycles - International Bipolar Foundation [Internet]. 2021 [cited 2025 Mar 24]. Available from: https://ibpf.org/seasons-and-cycles/.

[15] Bipolar UK [Internet]. 2019 [cited 2025 Mar 24]. Seasons and mood. Available from: https://www.bipolaruk.org/blog/seasons-and-mood.

[16] Wise D Bipolar Depression: Persevering Through the Winter Blues [Internet]. bpHope.com. 2015 [cited 2025 Mar 24]. Available from: https://www.bphope.com/blog/bipolar-winter-blues/.

[17] Small L, Lundell LS, Iversen J, Ehrlich AM, Dall M, Basse AL, et al. Seasonal light hours modulate peripheral clocks and energy metabolism in mice. Cell Metab. 2023;35:1722–1735.e5. 37689069 10.1016/j.cmet.2023.08.005

[18] Laakso ML, Porkka-Heiskanen T, Alila A, Stenberg D, Johansson G. Twenty-four- hour rhythms in relation to the natural photoperiod: a field study in humans. J Biol Rhythms. 1994;9:283–93. 7772796 10.1177/074873049400900309

[19] Körtner G, Geiser F. The temporal organization of daily torpor and hibernation: circadian and circannual rhythms. Chronobiol Int. 2000;17:103–28. 10757457 10.1081/cbi-100101036

[20] Geiser F, McAllan BM, Kenagy GJ, Hiebert SM. Photoperiod affects daily torpor and tissue faZy acid composition in deer mice. Naturwissenschaften. 2007;94:319–25. 17160415 10.1007/s00114-006-0193-z

[21] Van Doren BM, Liedvogel M, Helm B. Programmed and flexible: long-term Zugunruhe data highlight the many axes of variation in avian migratory behaviour. J Avian Biol. 2017;48:155–72.

[22] Gwinner E. Circannual clocks in avian reproduction and migration. Ibis. 1996;138:47–63.

[23] Dopico XC, Evangelou M, Ferreira RC, Guo H, Pekalski ML, Smyth DJ, et al. Widespread seasonal gene expression reveals annual differences in human immunity and physiology. Nat Commun. 2015;6:7000. 25965853 10.1038/ncomms8000 PMC4432600

[24] Drucker DJ. The GLP-1 journey: from discovery science to therapeutic impact. J Clin Invest. 2024;134:e175634. 38226625 10.1172/JCI175634 PMC10786682

[25] Quianzon CC, Cheikh I History of insulin. J Community Hosp Intern Med Perspect. 2012;2: 10.3402/jchimp.v2i2.18701. 10.3402/jchimp.v2i2.18701 PMC3714061 23882369

[26] Revel FG, Herwig A, Garidou ML, Dardente H, Menet JS, Masson-Pévet M, et al. The circadian clock stops ticking during deep hibernation in the European hamster. Proc Natl Acad Sci. 2007;104:13816–20. 17715068 10.1073/pnas.0704699104 PMC1959465

[27] WaZs AJ, Storey KB. Peripheral circadian gene activity is altered during hibernation in the thirteen-lined ground squirrel. Cryobiology. 2022;107:48–56. 35613673 10.1016/j.cryobiol.2022.05.003

[28] Andrews MT. Molecular interactions underpinning the phenotype of hibernation in mammals. J Exp Biol. 2019;222:jeb160606. 30683731 10.1242/jeb.160606

[29] Giroud S, Habold C, Nespolo RF, Mejías C, Terrien J, Logan SM, et al. The torpid state: recent advances in metabolic adaptations and protective mechanisms†. Front Physiol. 2021;11:623665. 33551846 10.3389/fphys.2020.623665 PMC7854925

[30] Hastings MH, Maywood ES, Brancaccio M. Generation of circadian rhythms in the suprachiasmatic nucleus. Nat Rev Neurosci. 2018;19:453–69. 29934559 10.1038/s41583-018-0026-z

[31] Heller HC, Ruby NF. Sleep and circadian rhythms in mammalian torpor. Annu Rev Physiol. 2004;66:275–89. 14977404 10.1146/annurev.physiol.66.032102.115313

[32] Gautier C, Bothorel B, Ciocca D, Valour D, Gaudeau A, Dupré C, et al. Gene expression profiling during hibernation in the European hamster. Sci Rep. 2018;8:13167. 30177816 10.1038/s41598-018-31506-2 PMC6120936

[33] Barinaga M. New timepiece has a familiar ring. Science. 1998;281:1429–30. 9750109 10.1126/science.281.5382.1429

[34] Mansour HA, Monk TH, Nimgaonkar VL. Circadian genes and bipolar disorder. Ann Med. 2005;37:196–205. 16019718 10.1080/07853890510007377

[35] Schuch JB, Genro JP, Bastos CR, Ghisleni G, Tovo-Rodrigues L. The role of CLOCK gene in psychiatric disorders: Evidence from human and animal research. Am J Med Genet B Neuropsychiatr Genet. 2018;177:181–98. 28902457 10.1002/ajmg.b.32599

[36] Nurnberger JI Jr, Adkins S, Lahiri DK, Mayeda A, Hu K, Lewy A, et al. Melatonin suppression by light in euthymic bipolar and unipolar patients. Arch Gen Psychiatry. 2000;57:572–9. 10839335 10.1001/archpsyc.57.6.572

[37] Melloni EMT, Paolini M, Dallaspezia S, Lorenzi C, PoleZi S, d’Orsi G, et al. Melatonin secretion paZerns are associated with cognitive vulnerability and brain structure in bipolar depression. Chronobiol Int. 2023;40:1279–90. 37781880 10.1080/07420528.2023.2262572

[38] Florant GL, Rivera ML, Lawrence AK, Tamarkin L. Plasma melatonin concentrations in hibernating marmots: absence of a plasma melatonin rhythm. Am J Physiol. 1984;247:R1062–1066. 6507651 10.1152/ajpregu.1984.247.6.R1062

[39] Vaněček J, Janský L, Illnerová H, Hoffmann K. Pineal melatonin in hibernating and aroused golden hamsters (*Mesocricetus auratus*). Comp Biochem Physiol A Physiol. 1984;77:759–62. 10.1016/0300-9629(84)90197-x 6143647

[40] RiZer P, Soltmann B, Sauer C, Yakac A, Boekstaegers L, Reichard M, et al. Supersensitivity of patients with bipolar I disorder to light-induced phase delay by narrow bandwidth blue light. Biol Psychiatry Glob Open Sci. 2021;2:28–35. 36324599 10.1016/j.bpsgos.2021.06.004 PMC9616289

[41] Burns AC, Windred DP, RuZer MK, Olivier P, VeZer C, Saxena R, et al. Day and night light exposure are associated with psychiatric disorders: an objective light study in >85,000 people. Nat Ment Health. 2023;1:853–62.

[42] Xu YX, Zhang JH, Ding WQ. Association of light at night with cardiometabolic disease: a systematic review and meta-analysis. Environ Pollut. 2024;342:123130. 38081378 10.1016/j.envpol.2023.123130

[43] Mokros L, Nowakowska-Domagała K, Witusik A, Pietras T. Evening chronotype as a bipolar feature among patients with major depressive disorder: the results of a pilot factor analysis. Braz J Psychiatry. 2021;44:35–40. 10.1590/1516-4446-2021-1747 PMC8827374 35170673

[44] Harvey AG, Talbot LS, Gershon A. Sleep disturbance in bipolar disorder across the lifespan. Clin Psychol Publ Div Clin Psychol Am Psychol Assoc. 2009;16:256–77. 10.1111/j.1468-2850.2009.01164.x PMC3321357 22493520

[45] Talih F, Gebara NY, Andary FS, Mondello S, Kobeissy F, Ferri R. Delayed sleep phase syndrome and bipolar disorder: Pathogenesis and available common biomarkers. Sleep Med Rev. 2018;41:133–40. 29534856 10.1016/j.smrv.2018.02.002

[46] Vancampfort D, Mitchell AJ, Hert MD, Sienaert P, Probst M, Buys R, et al. Prevalence and predictors of type 2 diabetes mellitus in people with bipolar disorder: a systematic review and meta-analysis. J Clin Psychiatry. 2015;76:15482. 10.4088/JCP.14r09635 26214054

[47] Barbieri M, Bonafè M, Franceschi C, Paolisso G. Insulin/IGF-I-signaling pathway: an evolutionarily conserved mechanism of longevity from yeast to humans. Am J Physiol Endocrinol Metab. 2003;285:E1064–1071. 14534077 10.1152/ajpendo.00296.2003

[48] Kimura KD, Tissenbaum HA, Liu Y, Ruvkun G. daf-2, an insulin receptor-like gene that regulates longevity and diapause in Caenorhabditis elegans. Science. 1997;277:942–6. 9252323 10.1126/science.277.5328.942

[49] Brogiolo W, Stocker H, Ikeya T, Rintelen F, Fernandez R, Hafen E. An evolutionarily conserved function of the Drosophila insulin receptor and insulin- like peptides in growth control. Curr Biol CB. 2001;11:213–21. 11250149 10.1016/s0960-9822(01)00068-9

[50] Nässel DR, Vanden Broeck J. Insulin/IGF signaling in Drosophila and other insects: factors that regulate production, release and post-release action of the insulin-like peptides. Cell Mol Life Sci CMLS. 2016;73:271–90. 26472340 10.1007/s00018-015-2063-3 PMC11108470

[51] Chan SJ, Steiner DF. Insulin through the ages: phylogeny of a growth promoting and metabolic regulatory hormone. Am Zool. 2000;40:213–22.

[52] Taniguchi CM, Emanuelli B, Kahn CR. Critical nodes in signalling pathways: insights into insulin action. Nat Rev Mol Cell Biol. 2006;7:85–96. 16493415 10.1038/nrm1837

[53] Maudsley H. The Pathology of Mind. London: Macmillan and Co; 1879.

[54] Wellcome Collection [Internet]. [cited 2025 Mar 30]. Insanity and allied neuroses : practical and clinical / by George H. Savage. Available from: https://wellcomecollection.org/works/mxq8hj93/items.

[55] Kretschmer E. Physique And Character [Internet]. Kegan Paul. Trench, Trubner And Company., Limited; 1925. p. 353 [cited 2025 Mar 30]Available from: http://archive.org/details/physiqueandchara031966mbp.

[56] van der Velde CD, Gordon MW. Manic-depressive illness, diabetes mellitus, and lithium carbonate. Arch Gen Psychiatry. 1969;21:478–85. 5807757 10.1001/archpsyc.1969.01740220094011

[57] Miola A, Alvarez-Villalobos NA, Ruiz-Hernandez FG, De Filippis E, Veldic M, Prieto ML, et al. Insulin resistance in bipolar disorder: a systematic review of illness course and clinical correlates. J Affect Disord. 2023;334:1–11. 37086806 10.1016/j.jad.2023.04.068

[58] McElroy SL, Keck PE. Metabolic syndrome in bipolar disorder: a review with a focus on bipolar depression. J Clin Psychiatry. 2014;75:46–61. 24502861 10.4088/JCP.13r08634

[59] Coello K, Vinberg M, Knop FK, Pedersen BK, McIntyre RS, Kessing LV, et al. Metabolic profile in patients with newly diagnosed bipolar disorder and their unaffected first-degree relatives. Int J Bipolar Disord. 2019;7:8–8. 30937579 10.1186/s40345-019-0142-3 PMC6443746

[60] Li K, Li T, Yang T, Lin Y, Liao Y, Gan Z. Prevalence of insulin resistance and its associated factors in drug-naïve patients with bipolar disorder among Han Chinese population. BMC Psychiatry. 2024;24:388. 38783222 10.1186/s12888-024-05838-5 PMC11112952

[61] Guha P, Bhowmick K, Mazumder P, Ghosal M, Chakraborty I, Burman P. Assessment of insulin resistance and metabolic syndrome in drug naive patients of bipolar disorder. Indian J Clin Biochem. 2014;29:51–6. 24478549 10.1007/s12291-012-0292-x PMC3903932

[62] Gao X, Qin Y, Jiao S, Hao J, Zhao J, Wang J, et al. Genetic evidence for the causal relations between metabolic syndrome and psychiatric disorders: a Mendelian randomization study. Transl Psychiatry. 2024;14:46. 38245519 10.1038/s41398-024-02759-5 PMC10799927

[63] Amare AT, Schubert KO, Klingler-Hoffmann M, Cohen-Woods S, Baune BT. The genetic overlap between mood disorders and cardiometabolic diseases: a systematic review of genome wide and candidate gene studies. Transl Psychiatry. 2017;7:e1007. 28117839 10.1038/tp.2016.261 PMC5545727

[64] Wray NR, Ripke S, MaZheisen M, Trzaskowski M, Byrne EM, Abdellaoui A, et al. Genome-wide association analyses identify 44 risk variants and refine the genetic architecture of major depression. Nat Genet. 2018;50:668–81. 29700475 10.1038/s41588-018-0090-3 PMC5934326

[65] Tessier SN, Zhang J, Biggar KK, Wu CW, Pifferi F, Perret M, et al. Regulation of the PI3K/AKT pathway and fuel utilization during primate torpor in the gray mouse lemur, microcebus murinus. Genomics Proteomics Bioinformatics. 2015;13:91–102. 26092184 10.1016/j.gpb.2015.03.006 PMC4511781

[66] Green SR, Al-AZar R, McKechnie AE, Naidoo S, Storey KB. Role of Akt signaling pathway regulation in the speckled mousebird (*Colius striatus*) during torpor displays tissue specific responses. Cell Signal. 2020;75:109763. 32871209 10.1016/j.cellsig.2020.109763

[67] Rogalska J, Caputa M Hypometabolism as a strategy of survival in asphyxiated newborn mammals. In 2011. p. 117–45.

[68] Green SR, Al-AZar R, McKechnie AE, Naidoo S, Storey KB. Phosphorylation status of pyruvate dehydrogenase in the mousebird Colius striatus undergoing torpor. J Exp Zool Part Ecol Integr Physiol. 2022;337:337–45. 10.1002/jez.2570 34951526

[69] Wijenayake S, Luu BE, Zhang J, Tessier SN, Quintero-Galvis JF, Gaitán-Espitia JD, et al. Strategies of biochemical adaptation for hibernation in a South American marsupial, *Dromiciops gliroides*: 4. Regulation of pyruvate dehydrogenase complex and metabolic fuel selection. Comp Biochem Physiol B Biochem Mol Biol. 2018;224:32–7. 29247844 10.1016/j.cbpb.2017.12.008

[70] Campbell IH, Campbell H, Smith DJ. Insulin signaling as a therapeutic mechanism of lithium in bipolar disorder. Transl Psychiatry. 2022;12:350. 36038539 10.1038/s41398-022-02122-6 PMC9424309

[71] Campbell I, Campbell H. Mechanisms of insulin resistance, mitochondrial dysfunction and the action of the ketogenic diet in bipolar disorder. Focus on the PI3K/AKT/HIF1-a pathway. Med Hypotheses. 2020;145:110299. 33091780 10.1016/j.mehy.2020.110299

[72] Beurel E, Grieco SF, Jope RS. Glycogen synthase kinase-3 (GSK3): regulation, actions, and diseases. Pharmacol Ther. 2015;148:114–31. 25435019 10.1016/j.pharmthera.2014.11.016 PMC4340754

[73] Freland L, Beaulieu JM. Inhibition of GSK3 by lithium, from single molecules to signaling networks. Front Mol Neurosci. 2012;5:14. 22363263 10.3389/fnmol.2012.00014 PMC3282483

[74] Ryves WJ, Harwood AJ. Lithium inhibits glycogen synthase kinase-3 by competition for magnesium. Biochem Biophys Res Commun. 2001;280:720–5. 11162580 10.1006/bbrc.2000.4169

[75] Tabata I, Schluter J, Gulve EA, Holloszy JO. Lithium increases susceptibility of muscle glucose transport to stimulation by various agents. Diabetes. 1994;43:903–7. 8013755 10.2337/diab.43.7.903

[76] Gherardelli C, Cisternas P, Inestrosa NC. Lithium enhances hippocampal glucose metabolism in an in vitro mice model of Alzheimer’s disease. Int J Mol Sci. 2022;23:8733. 35955868 10.3390/ijms23158733 PMC9368914

[77] Kohno T, Shiga T, Toyomaki A, Kusumi I, Matsuyama T, Inoue T, et al. Effects of lithium on brain glucose metabolism in healthy men. J Clin Psychopharmacol. 2007;27:698. 18004140 10.1097/jcp.0b013e31815a23c2

[78] Khayachi A, Abuzgaya M, Liu Y, Jiao C, Dejgaard K, Schorova L, et al. Akt and AMPK activators rescue hyperexcitability in neurons from patients with bipolar disorder. EBioMedicine. 2024;104:105161. 38772282 10.1016/j.ebiom.2024.105161 PMC11134542

[79] Jensen J, Rustad PI, Kolnes AJ, Lai YC. The role of skeletal muscle glycogen breakdown for regulation of insulin sensitivity by exercise. Front Physiol. 2011;2:112. 22232606 10.3389/fphys.2011.00112 PMC3248697

[80] Siri WE. The gross composition of the body. Adv Biol Med Phys. 1956;4:239–80. 13354513 10.1016/b978-1-4832-3110-5.50011-x

[81] Colagiuri S, Miller JB. The ‘carnivore connection’—evolutionary aspects of insulin resistance. Eur J Clin Nutr. 2002;56:S30–5. 11965520 10.1038/sj.ejcn.1601351

[82] Lockhart SM, Saudek V, O’Rahilly S. GDF15: a hormone conveying somatic distress to the brain. Endocr Rev. 2020;41:bnaa007. 32310257 10.1210/endrev/bnaa007 PMC7299427

[83] Hsu JY, Crawley S, Chen M, Ayupova DA, Lindhout DA, Higbee J, et al. Non- homeostatic body weight regulation through a brainstem-restricted receptor for GDF15. Nature. 2017;550:255–9. 28953886 10.1038/nature24042

[84] Paul JR, McKeown AS, Davis JA, Totsch SK, Minã EM, Kraft TW, et al. Glycogen synthase kinase 3 regulates photic signaling in the suprachiasmatic nucleus. Eur J Neurosci. 2017;45:1102–10. 28244152 10.1111/ejn.13549 PMC5395359

[85] Iitaka C, Miyazaki K, Akaike T, Ishida N. A role for glycogen synthase kinase- 3beta in the mammalian circadian clock. J Biol Chem. 2005;280:29397–402. 15972822 10.1074/jbc.M503526200

[86] Tessier SN, Wu CW, Storey KB. Molecular control of protein synthesis, glucose metabolism, and apoptosis in the brain of hibernating thirteen-lined ground squirrels. Biochem Cell Biol. 2019;97:536–44. 30763120 10.1139/bcb-2018-0256

[87] Lee YJ, Bernstock JD, Klimanis D, Hallenbeck JM. Akt protein kinase, miR- 200/miR-182 expression and epithelial-mesenchymal transition proteins in hibernating ground squirrels. Front Mol Neurosci. 2018;11:22. 29440989 10.3389/fnmol.2018.00022 PMC5797618

[88] Li X, Liu M, Cai Z, Wang G, Li X. Regulation of glycogen synthase kinase-3 during bipolar mania treatment. Bipolar Disord. 2010;12:741–52. 21040291 10.1111/j.1399-5618.2010.00866.x PMC3059222

[89] Tye SJ, Borreggine K, Price JB, Sutor SL, Cuéllar-Barboza AB, McElroy SL, et al. Dynamic insulin-stimulated mTOR/GSK3 signaling in peripheral immune cells: Preliminary evidence for an association with lithium response in bipolar disorder. Bipolar Disord. 2022;24:39–47. 33864716 10.1111/bdi.13081

[90] Rosso G, Maina G, Teobaldi E, Balbo I, Di Salvo G, Montarolo F, et al. Differential diagnosis of unipolar versus bipolar depression by GSK3 levels in peripheral blood: a pilot experimental study. Int J Bipolar Disord. 2023;11:33. 37807001 10.1186/s40345-023-00314-7 PMC10560641

[91] Wu CW, Storey KB. Regulation of the mTOR signaling network in hibernating thirteen-lined ground squirrels. J Exp Biol. 2012;215:1720–7. 22539739 10.1242/jeb.066225

[92] Machado-Vieira R, ZaneZi MV, Teixeira AL, Uno M, Valiengo LL, Soeiro-de- Souza MG, et al. Decreased AKT1/mTOR pathway mRNA expression in short- term bipolar disorder. Eur Neuropsychopharmacol. 2015;25:468–73. 25726893 10.1016/j.euroneuro.2015.02.002 PMC5863235

[93] Vanderplow AM, Eagle AL, Kermath BA, Bjornson KJ, Robison AJ, Cahill ME. Akt-mTOR hypoactivity in bipolar disorder gives rise to cognitive impairments associated with altered neuronal structure and function. Neuron. 2021;109:1479. 33765445 10.1016/j.neuron.2021.03.008 PMC8105282

[94] Laplante M, Sabatini DM. mTOR signaling in growth control and disease. Cell. 2012;149:274–93. 22500797 10.1016/j.cell.2012.03.017 PMC3331679

[95] Kim SH, Yu HS, Park HG, Ha K, Kim YS, Shin SY, et al. Intracerebroventricular administration of ouabain, a Na/K-ATPase inhibitor, activates mTOR signal pathways and protein translation in the rat frontal cortex. Prog Neuropsychopharmacol Biol Psychiatry. 2013;45:73–82. 23643758 10.1016/j.pnpbp.2013.04.018

[96] Kara NZ, Flaisher-Grinberg S, Anderson GW, Agam G, Einat H. Mood- stabilizing effects of rapamycin and its analog temsirolimus: relevance to autophagy. Behav Pharmacol. 2018;29:379. 28777104 10.1097/FBP.0000000000000334

[97] Chen Y, Guan W, Wang ML, Lin XY. PI3K-AKT/mTOR signaling in psychiatric disorders: a valuable target to stimulate or suppress? Int J Neuropsychopharmacol. 2024;27:pyae010. 38365306 10.1093/ijnp/pyae010 PMC10888523

[98] Abdallah CG, Averill LA, Gueorguieva R, Goktas S, Purohit P, Ranganathan M, et al. Modulation of the antidepressant effects of ketamine by the mTORC1 inhibitor rapamycin. Neuropsychopharmacology. 2020;45:990–7. 32092760 10.1038/s41386-020-0644-9 PMC7162891

[99] Kamata T, Yamada S, Sekijima T. Differential AMPK-mediated metabolic regulation observed in hibernation-style polymorphisms in Siberian chipmunks. Front Physiol. 2023;14:1220058. 37664438 10.3389/fphys.2023.1220058 PMC10468594

[100] Yamada S, Kamata T, Nawa H, Sekijima T, Takei N. AMPK activation, eEF2 inactivation, and reduced protein synthesis in the cerebral cortex of hibernating chipmunks. Sci Rep. 2019;9:11904. 31417118 10.1038/s41598-019-48172-7 PMC6695389

[101] Lamia KA, Sachdeva UM, DiTacchio L, Williams EC, Alvarez JG, Egan DF, et al. AMPK regulates the circadian clock by cryptochrome phosphorylation and degradation. Science. 2009;326:437–40. 19833968 10.1126/science.1172156 PMC2819106

[102] Calkin CV, Chengappa KNR, Cairns K, Cookey J, Gannon J, Alda M, et al. Treating insulin resistance with metformin as a strategy to improve clinical outcomes in treatment-resistant bipolar depression (the TRIO-BD Study): a randomized, quadruple-masked, placebo-controlled clinical trial. J Clin Psychiatry. 2022;83:21m14022. 35120288 10.4088/JCP.21m14022

[103] Dilliraj LN, Schiuma G, Lara D, Strazzabosco G, Clement J, Giovannini P, et al. The evolution of ketosis: potential impact on clinical conditions. Nutrients. 2022;14:3613. 36079870 10.3390/nu14173613 PMC9459968

[104] Rauch JC. Ketone bodies: a source of energy during hibernation. Can J Zool. 1981;59:754–60.

[105] Krilowicz BL. Ketone body metabolism in a ground squirrel during hibernation and fasting. Am J Physiol. 1985;249:R462–470. 4051032 10.1152/ajpregu.1985.249.4.R462

[106] Andrews MT, Russeth KP, Drewes LR, Henry PG. Adaptive mechanisms regulate preferred utilization of ketones in the heart and brain of a hibernating mammal during arousal from torpor. Am J Physiol - Regul Integr Comp Physiol. 2009;296:R383–93. 19052316 10.1152/ajpregu.90795.2008 PMC2643978

[107] Veech RL. The therapeutic implications of ketone bodies: the effects of ketone bodies in pathological conditions: ketosis, ketogenic diet, redox states, insulin resistance, and mitochondrial metabolism. Prostaglandins Leukot Essent FaZy Acids. 2004;70:309–19. 10.1016/j.plefa.2003.09.007 14769489

[108] Sofou K, Dahlin M, Hallböök T, Lindefeldt M, Viggedal G, Darin N. Ketogenic diet in pyruvate dehydrogenase complex deficiency: short- and long-term outcomes. J Inherit Metab Dis. 2017;40:237–45. 28101805 10.1007/s10545-016-0011-5 PMC5306430

[109] Campbell I, Campbell H. A pyruvate dehydrogenase complex disorder hypothesis for bipolar disorder. Med Hypotheses. 2019;130:109263. 31383331 10.1016/j.mehy.2019.109263

[110] Kim JM. Ketogenic diet: old treatment, new beginning. Clin Neurophysiol Pract. 2017;2:161. 30214990 10.1016/j.cnp.2017.07.001 PMC6123870

[111] Martin-McGill KJ, Bresnahan R, Levy RG, Cooper PN. Ketogenic diets for drug-resistant epilepsy. Cochrane Database Syst Rev. 2020;6:CD001903. 32588435 10.1002/14651858.CD001903.pub5 PMC7387249

[112] Saltiel AR. Insulin signaling in health and disease. J Clin Invest. 2021;131:e142241. 33393497 10.1172/JCI142241 PMC7773347

[113] Campbell IH, Needham N, Grossi H, Kamenska I, Luz S, Sheehan S, et al. A pilot study of a ketogenic diet in bipolar disorder: clinical, metabolic and magnetic resonance spectroscopy findings. BJPsych Open. 2025;11:e34. 39995103 10.1192/bjo.2024.841 PMC12001942

[114] Sethi S, Wakeham D, KeZer T, Hooshmand F, Bjornstad J, Richards B, et al. Ketogenic diet intervention on metabolic and psychiatric health in bipolar and schizophrenia: a pilot trial. Psychiatry Res. 2024;335:115866. 38547601 10.1016/j.psychres.2024.115866

[115] Danan A, Westman EC, Saslow LR, Ede G. The ketogenic diet for refractory mental illness: a retrospective analysis of 31 inpatients. Front Psychiatry. 2022;13:951376. 35873236 10.3389/fpsyt.2022.951376 PMC9299263

[116] Campbell IH, Campbell H. Ketosis and bipolar disorder: controlled analytic study of online reports. BJPsych Open. 2019;5:e58. 31530294 10.1192/bjo.2019.49 PMC6620566

[117] Chmiel I. Ketogenic diet in therapy of bipolar affective disorder - case report and literature review. Psychiatr Pol. 2022;56:1345–63. 37098202 10.12740/PP/OnlineFirst/136356

[118] Phelps JR, Siemers SV, El-Mallakh RS. The ketogenic diet for type II bipolar disorder. Neurocase. 2013;19:423–6. 23030231 10.1080/13554794.2012.690421

[119] Palmer CM, Gilbert-Jaramillo J, Westman EC. The ketogenic diet and remission of psychotic symptoms in schizophrenia: two case studies. Schizophr Res. 2019;208:439–40. 30962118 10.1016/j.schres.2019.03.019

[120] Laurent N, Bellamy EL, Tague KA, Hristova D, Houston A. Ketogenic metabolic therapy for schizoaffective disorder: a retrospective case series of psychotic symptom remission and mood recovery. Front Nutr. 2025;12:1506304. 39990610 10.3389/fnut.2025.1506304 PMC11844221

[121] Wehr TA. Melatonin and seasonal rhythms. J Biol Rhythms. 1997;12:518–27. 9406025 10.1177/074873049701200605

[122] Vella CA, Nelson OL, Jansen HT, Robbins CT, Jensen AE, Constantinescu S, et al. Regulation of metabolism during hibernation in brown bears (Ursus arctos): Involvement of cortisol, PGC-1α and AMPK in adipose tissue and skeletal muscle. Comp Biochem Physiol A Mol Integr Physiol. 2020;240:110591. 31669707 10.1016/j.cbpa.2019.110591

[123] Milo T, Maimon L, Cohen B, Haran D, Segman D, Danon T, et al. Longitudinal hair cortisol in bipolar disorder and a mechanism based on HPA dynamics. iScience. 2024;27:109234. 38482495 10.1016/j.isci.2024.109234 PMC10933461

[124] Bouma HR, Carey HV, Kroese FGM. Hibernation: the immune system at rest? J Leukoc Biol. 2010;88:619–24. 20519639 10.1189/jlb.0310174

[125] Munkholm K, Vinberg M, Vedel Kessing L. Cytokines in bipolar disorder: a systematic review and meta-analysis. J Affect Disord. 2013;144:16–27. 22749156 10.1016/j.jad.2012.06.010

[126] Dallaspezia S, Cardaci V, Mazza MG, De Lorenzo R, Rovere Querini P, Colombo C, et al. Higher seasonal variation of systemic inflammation in bipolar disorder. Int J Mol Sci. 2024;25:4310. 38673894 10.3390/ijms25084310 PMC11049938

[127] Dickerson F, Stallings C, Origoni A, Vaughan C, Katsafanas E, Khushalani S, et al. A Combined Marker of Inflammation in Individuals with Mania. PLOS ONE. 2013;8:e73520. 24019926 10.1371/journal.pone.0073520 PMC3760815

[128] Lu YR, Rao YB, Mou YJ, Chen Y, Lou HF, Zhang Y, et al. High concentrations of serum interleukin-6 and interleukin-8 in patients with bipolar disorder. Medicine (Baltimore). 2019;98:e14419. 30762747 10.1097/MD.0000000000014419 PMC6407988

[129] Ralat SI, Martinez K, Rodriguez RJ, Gerena Y. Team the IMP. 357 inflammatory cytokines and neurocognitive functioning in bipolar patients across mood episodes. J Clin Transl Sci. 2022;6:66–7.

[130] Wu CW, Biggar KK, Storey KB. Biochemical adaptations of mammalian hibernation: exploring squirrels as a perspective model for naturally induced reversible insulin resistance. Braz J Med Biol Res. 2013;46:1–13. 23314346 10.1590/1414-431X20122388 PMC3854349

[131] Chazarin B, Storey KB, Ziemianin A, Chanon S, Plumel M, Chery I, et al. Metabolic reprogramming involving glycolysis in the hibernating brown bear skeletal muscle. Front Zool. 2019;16:12. 31080489 10.1186/s12983-019-0312-2 PMC6503430

[132] Mansur RB, Rizzo LB, Santos CM, Asevedo E, Cunha GR, Noto MN, et al. Impaired glucose metabolism moderates the course of illness in bipolar disorder. J Affect Disord. 2016;195:57–62. 26866976 10.1016/j.jad.2016.02.002

[133] Wu C, Ren C, Teng Z, Li S, Silva F, Wu H, et al. Cerebral glucose metabolism in bipolar disorder: A voxel-based meta-analysis of positron emission tomography studies. Brain Behav. 2021;11:e02117. 33769704 10.1002/brb3.2117 PMC8119802

[134] Staples JF. Metabolic suppression in mammalian hibernation: the role of mitochondria. J Exp Biol. 2014;217:2032–6. 24920833 10.1242/jeb.092973

[135] Baxter LR, Phelps ME, MazzioZa JC, Schwarã JM, Gerner RH, Selin CE, et al. Cerebral metabolic rates for glucose in mood disorders. Studies with positron emission tomography and fluorodeoxyglucose F 18. Arch Gen Psychiatry. 1985;42:441–7. 3872649 10.1001/archpsyc.1985.01790280019002

[136] Miranda-PeZersen K, Bezerra-Filho S, Pinheiro TB, Oliva-Costa SF, Miranda-Scippa Â. Is there a relationship between physical activity and residual mood symptoms in patients with bipolar I disorder? Ment Health Phys Act. 2020;19:100352.

[137] Miyazaki M, Shimozuru M, Kitaoka Y, Takahashi K, Tsubota T. Regulation of protein and oxidative energy metabolism are down-regulated in the skeletal muscles of Asiatic black bears during hibernation. Sci Rep. 2022;12:19723. 36385156 10.1038/s41598-022-24251-0 PMC9668988

[138] Rowe MK, Wiest C, Chuang DM. GSK-3 is a viable potential target for therapeutic intervention in bipolar disorder. Neurosci Biobehav Rev. 2007;31:920–31. 17499358 10.1016/j.neubiorev.2007.03.002 PMC2020444

[139] Rouble AN, Storey KB. Characterization of the SIRT family of NAD+- dependent protein deacetylases in the context of a mammalian model of hibernation, the thirteen-lined ground squirrel. Cryobiology. 2015;71:334–43. 26277038 10.1016/j.cryobiol.2015.08.009

[140] Alageel A, Tomasi J, Tersigni C, Brieãke E, Zuckerman H, Subramaniapillai M, et al. Evidence supporting a mechanistic role of sirtuins in mood and metabolic disorders. Prog Neuropsychopharmacol Biol Psychiatry. 2018;86:95–101. 29802856 10.1016/j.pnpbp.2018.05.017

[141] Abe N, Uchida S, Otsuki K, Hobara T, Yamagata H, Higuchi F, et al. Altered sirtuin deacetylase gene expression in patients with a mood disorder. J Psychiatr Res. 2011;45:1106–12. 21349544 10.1016/j.jpsychires.2011.01.016

[142] Belvederi Murri M, Prestia D, Mondelli V, Pariante C, PaZi S, Olivieri B, et al. The HPA axis in bipolar disorder: Systematic review and meta-analysis. Psychoneuroendocrinology. 2016;63:327–42. 26547798 10.1016/j.psyneuen.2015.10.014

[143] Mukherjee D, Weissenkampen JD, Wasserman E, Krishnamurthy VB, MilleZ CE, Conway S, et al. Dysregulated diurnal cortisol PaZern and heightened night-time cortisol in individuals with bipolar disorder. Neuropsychobiology. 2022;81:51–9. 34320487 10.1159/000517343 PMC8795243

[144] Li X, Yu J, Jiang S, Fang L, Li Y, Ma S, et al. Circadian rhythms of melatonin and its relationship with anhedonia in patients with mood disorders: a cross- sectional study. BMC Psychiatry. 2024;24:165. 38413912 10.1186/s12888-024-05606-5 PMC10900661

[145] Palumbo PJ, Wellik DL, Bagley NA, Nelson RA. Insulin and glucagon responses in the hibernating black bear. Bears Their Biol Manag. 1983;5:291–6.

[146] Frøbert AM, Nielsen CG, Brohus M, Kindberg J, Fröbert O, Overgaard MT. Hypothyroidism in hibernating brown bears. Thyroid Res. 2023;16:3. 36721203 10.1186/s13044-022-00144-2 PMC9890737

[147] Chakrabarti S. Thyroid functions and bipolar affective disorder. J Thyroid Res. 2011;2011:306367. 21808723 10.4061/2011/306367 PMC3144691

[148] Barbosa IG, Machado-Vieira R, Soares JC, Teixeira AL. The immunology of bipolar disorder. Neuroimmunomodulation. 2014;21:117–22. 24557044 10.1159/000356539 PMC4041530

[149] Tessier SN, Kaãenback BA, Pifferi F, Perret M, Storey KB. Cytokine and antioxidant regulation in the intestine of the gray mouse lemur (Microcebus Murinus) during torpor. Genomics Proteomics Bioinformatics. 2015;13:127–35. 26092185 10.1016/j.gpb.2015.03.005 PMC4511783

[150] Luo Y, He H, Zhang M, Huang X, Fan N. Altered serum levels of TNF-*α*, IL-6 and IL-18 in manic, depressive, mixed state of bipolar disorder patients. Psychiatry Res. 2016;244:19–23. 27455146 10.1016/j.psychres.2016.07.027

[151] Novoselova EG, Kolaeva SG, Makar VR, Agaphonova TA. Production of tumor necrosis factor in cells of hibernating ground squirrels Citellus undulatus during annual cycle. Life Sci. 2000;67:1073–80. 10954040 10.1016/s0024-3205(00)00698-6

[152] Skibinska M, Rajewska-Rager A, Dmitrzak-Weglarz M, Kapelski P, Lepczynska N, Kaczmarek M, et al. Interleukin-8 and tumor necrosis factor-alpha in youth with mood disorders-A longitudinal study. Front Psychiatry. 2022;13:964538. 36032249 10.3389/fpsyt.2022.964538 PMC9403049

[153] Williams CT, Radonich M, Barnes BM, Buck CL. Seasonal loss and resumption of circadian rhythms in hibernating arctic ground squirrels. J Comp Physiol B. 2017;187:693–703. 28332018 10.1007/s00360-017-1069-6

[154] Kaufmann CN, Gershon A, Depp CA, Miller S, Zeiãer JM, KeZer TA. Daytime midpoint as a digital biomarker for chronotype in bipolar disorder. J Affect Disord. 2018;241:586–91. 30172210 10.1016/j.jad.2018.08.032 PMC6436809

[155] Gershon A, Kaufmann CN, Depp CA, Miller S, Do D, Zeiãer JM, et al. Subjective versus objective evening chronotypes in bipolar disorder. J Affect Disord. 2018;225:342–9. 28843917 10.1016/j.jad.2017.08.055 PMC5626649

[156] Shi J, WiZke-Thompson JK, Badner JA, HaZori E, Potash JB, Willour VL, et al. Clock genes may influence bipolar disorder susceptibility and dysfunctional circadian rhythm. Am J Med Genet Part B Neuropsychiatr Genet Off Publ Int Soc Psychiatr Genet. 2008;147B:1047–55. 10.1002/ajmg.b.30714 PMC2574897 18228528

[157] De Crescenzo F, Economou A, Sharpley AL, Gormez A, Quested DJ. Actigraphic features of bipolar disorder: a systematic review and meta-analysis. Sleep Med Rev. 2017 Jun;33:58–69. 28185811 10.1016/j.smrv.2016.05.003

[158] Buyukdura JS, McClintock SM, Croarkin PE. Psychomotor retardation in depression: Biological underpinnings, measurement, and treatment. Prog Neuropsychopharmacol Biol Psychiatry. 2011;35:395–409. 21044654 10.1016/j.pnpbp.2010.10.019 PMC3646325

[159] BenedeZi F, SerreZi A, Colombo C, Barbini B, Lorenzi C, Campori E, et al. Influence of CLOCK gene polymorphism on circadian mood fluctuation and illness recurrence in bipolar depression. Am J Med Genet Part B Neuropsychiatr Genet Off Publ Int Soc Psychiatr Genet. 2003;123B:23–6. 10.1002/ajmg.b.20038 14582141

[160] Mansour HA, Wood J, Logue T, Chowdari KV, Dayal M, Kupfer DJ, et al. Association study of eight circadian genes with bipolar I disorder, schizoaffective disorder and schizophrenia. Genes Brain Behav. 2006;5:150–7. 16507006 10.1111/j.1601-183X.2005.00147.x

[161] Morin P, Ni Z, McMullen DC, Storey KB. Expression of Nrf2 and its downstream gene targets in hibernating 13-lined ground squirrels, Spermophilus tridecemlineatus. Mol Cell Biochem. 2008;312:121–9. 18327701 10.1007/s11010-008-9727-3

[162] Fahey L, Lopez LM. Shared Genetic Links Between Sleep, Neurodevelopmental and Neuropsychiatric Conditions: A Genome-Wide and Pathway-Based Polygenic Score Analysis. Genes Brain Behav. 2024;23:e70011. 10.1111/gbb.70011 PMC11669943 39723615

[163] Wu CW, Storey KB. FoxO3a-mediated activation of stress responsive genes during early torpor in a mammalian hibernator. Mol Cell Biochem. 2014;390:185–95. 24493314 10.1007/s11010-014-1969-7

[164] Magno LAV, Santana CVN, Sacramento EK, Rezende VB, Cardoso MV, Maurício-da-Silva L, et al. Genetic variations in FOXO3A are associated with Bipolar Disorder without confering vulnerability for suicidal behavior. J Affect Disord. 2011;133:633–7. 21621268 10.1016/j.jad.2011.04.031

[165] Han Y, Zheng G, Yang T, Zhang S, Dong D, Pan YH. Adaptation of peroxisome proliferator-activated receptor alpha to hibernation in bats. BMC Evol Biol. 2015;15:88. 25980933 10.1186/s12862-015-0373-6 PMC4435907

[166] Zandi PP, Belmonte PL, Willour VL, Goes FS, Badner JA, Simpson SG, et al. Association study of Wnt signaling pathway genes in bipolar disorder. Arch Gen Psychiatry. 2008;65:785–93. 18606951 10.1001/archpsyc.65.7.785 PMC3170992

[167] Kasak M, Ceylan MF, Hesapcioglu ST, Senat A, Erel Ö. Peroxisome proliferator-activated receptor gamma (PPARγ) levels in adolescent with bipolar disorder and their relationship with metabolic parameters. J Mol Neurosci MN. 2022;72:1313–21. 35318563 10.1007/s12031-022-02000-2

[168] Inagaki T, Dutchak P, Zhao G, Ding X, Gautron L, Parameswara V, et al. Endocrine regulation of the fasting response by PPARalpha-mediated induction of fibroblast growth factor 21. Cell Metab. 2007;5:415–25. 17550777 10.1016/j.cmet.2007.05.003

[169] Chang HH, Chen PS, Cheng YW, Wang TY, Yang YK, Lu RB. FGF21 is associated with metabolic effects and treatment response in depressed bipolar II disorder patients treated with valproate. Int J Neuropsychopharmacol. 2017;21:319–24. 10.1093/ijnp/pyx093 PMC5888470 29618013

[170] Maistrovski Y, Biggar KK, Storey KB. HIF-1α regulation in mammalian hibernators: role of non-coding RNA in HIF-1α control during torpor in ground squirrels and bats. J Comp Physiol [B]. 2012;182:849–59. 10.1007/s00360-012-0662-y 22526261

[171] Shibata T, Yamagata H, Uchida S, Otsuki K, Hobara T, Higuchi F, et al. The alteration of hypoxia inducible factor-1 (HIF-1) and its target genes in mood disorder patients. Prog Neuropsychopharmacol Biol Psychiatry. 2013;43:222–9. 23333658 10.1016/j.pnpbp.2013.01.003

